# The psychosocial self‐efficacy in adolescents with type 1 diabetes

**DOI:** 10.1002/nop2.235

**Published:** 2019-01-17

**Authors:** Anne Survonen, Sanna Salanterä, Kirsti Näntö‐Salonen, Arun K. Sigurdardottir, Riitta Suhonen

**Affiliations:** ^1^ Department of Nursing Science, Turku University Hospital University of Turku Turku Finland; ^2^ Turku University Hospital Turku Finland; ^3^ School of Health Sciences University of Akureyri Akureyri Iceland; ^4^ Akureyri Hospital Akureyri Iceland; ^5^ Department of Nursing Science University of Turku and Turku University Hospital, and City of Turku, Welfare Division Turku Finland

**Keywords:** adolescent, diabetes, Diabetes Empowerment Scale, empowerment, psychometric properties, reliability, self‐efficacy, validity

## Abstract

**Aim:**

To analyse psychosocial self‐efficacy in adolescents with type 1 diabetes, evaluate associations between self‐efficacy and metabolic control and background variables and determine psychometric properties of the Finnish Diabetes Empowerment Scale (Fin‐DES‐28).

**Design:**

A descriptive correlational survey.

**Methods:**

The data were collected with the Finnish Diabetes Empowerment Scale from 13–16‐year‐old adolescents with type 1 diabetes (*N* = 189, 34%) in one university hospital district area in 2014.

**Results:**

The level of psychosocial self‐efficacy was quite good. The highest scores were in managing the psychosocial aspects of diabetes, followed by assessing dissatisfaction and readiness to change and setting and achieving diabetes goals. The self‐efficacy did not correlate with metabolic control or background variables. A positive association was found between self‐efficacy and understanding of diabetes and its treatment, adjustment of diabetes to life and the relationship with the doctor and the nurse. The internal consistency of the Finnish Diabetes Empowerment Scale was adequate. The low response rate limits generalization.

## 1 INTRODUCTION

Worldwide, approximately half a million children under 15 years old have type 1 diabetes (T1DM; Patterson et al., 2014) and about 86,000 children develop the disease each year (IDF, 2015). The highest incidence in the world is in Finland (IDF, 2015) where approximately 600 children and adolescents fall ill every year (Harjutsalo, Sjöberg, & Tuomilehto, 2008).

Type 1 diabetes is a long‐term condition requiring plenty of self‐care activities: monitoring blood glucose levels and administering insulin, diet and exercise (Khardori, 2017). Chronically ill adolescents face the same developmental issues as their healthy peers. Living with a long‐term illness may complicate normal development (Taylor et al., 2008). Diabetes may increase depression, anxiety and psychological distress as adolescents with T1DM have greater responsibility for their lives than their healthy peers (Buchberger et al., 2016; Delamater, Wit, McDarby, Malik, & Acerini, 2014). Symptoms of depression and anxiety may potentially compromise diabetes management and metabolic control (Buchberger et al., 2016). Adolescents may have problems with self‐care and adherence to diabetes regimens (Borus & Laffel, 2010).

Diabetes education of children and adolescents and their parents with newly diagnosed T1DM is based on a treatment plan made in every unit and is carried out by a multi‐professional team with a doctor, nurse, nutritionist, social worker/rehabilitation counsellor and psychiatric nurse (Current Care guideline for Insulin Deficiency Diabetes in Finland, 2018). At first, diabetes often fills the child's and adolescent's and their parents’ whole life. Gradually, the aim is to adapt diabetes management routines to children's and adolescents’ and their parents’ everyday life and to find a good balance with diabetes management and relaxedness to the life. Healthcare technology with insulin pumps and glucose sensors may make life with T1DM easier (Finnish Diabetes Association, 2018).

Diabetes education is the key to successful management of the illness, and it is recommended that educational interventions be based on clear theoretical psycho‐educational principles (Lange, Swift, Pankowska, & Danne, 2014). Educational interventions with indirect behavioural skill development facilitating diabetes management may improve the quality of life in adolescents with T1DM (Abualula, Jacobsen, Milligan, Rodan, & Conn, 2016). The aim of diabetes education is to support the adolescents and their parents to become empowered in diabetes management (Funnell & Anderson, 2003; Lange et al., 2014). Diabetes education needs to be adapted to adolescents’ age and level of development, learning ability (Lange et al., 2014) and their cognitive, biophysical, psychological and social needs. In addition, the values, beliefs and opinions of the adolescents need to be respected and taken into account in diabetes care (Funnell et al., 1991; Lange et al., 2014). Effective and empowering education can enhance adolescents’ perceived self‐efficacy in diabetes management (Anderson, Funnell, Fitzgerald, & Marrero, 2000) and should result in an ability to make informed decisions (Anderson & Funnell, 2010).

In Sweden, Viklund et al. (2007) studied the effects of an empowerment programme on glycaemic control and empowerment and the role of parental involvement in adolescents with T1DM. The empowerment programme had no effect on empowerment or glycaemic control except in the adolescents in one group whose parents were involved in the programme. In the study of Hanberger, Ludvigsson, and Nordfeldt (2013), the Diabit Web 2.0 net portal had no effect on empowerment. The findings differ from studies in adults. Internet‐based interventions (McCarrier et al., 2009; Samoocha et al., 2010), an intensive self‐management course (Lowe et al., 2008) and a brief (2.5 days) psycho‐educational intervention (George et al., 2008) had a positive impact on empowerment in adults with diabetes measured with the Diabetes Empowerment Scale.

In Finland, the studies of self‐management of T1DM in adolescents have focused on the concepts of compliance and adherence (Kyngäs, 2000; 2007; Kyngäs & Rissanen, 2001). The compliance‐oriented approach views patients as recipients of medical decisions and prescriptions whereas the empowerment‐oriented approach emphasizes patients being responsible for their choices (Aujoulat, d'Hoore, & Deccache, 2007). There is a need for a different approach in adolescents’ diabetes care. One Finnish study by Kelo, Eriksson, and Eriksson (2013) was found using empowering patient education for an intervention of blood glucose monitoring for elementary school‐aged children with T1DM and their parents. Nurses described the successful process of empowering patient education in blood glucose monitoring which consisted of need assessment, planning, implement and evaluation and faced some challenges related to ambivalence with traditional and empowering patient education (Kelo et al., 2013).

### 1.1 Background

Self‐efficacy is an individual's judgement of his or her ability to organize and execute courses of action and is task‐ and context‐specific (Bandura, 1997). Self‐efficacy determines the behaviour of patients with long‐term diseases. Adolescents with a sense of self‐efficacy are more confident in disease management, which can benefit their physical ability (Resnick, 2014) and may result in positive outcome expectations (Newton & Ashley, 2013) and a higher probability of achieving target metabolic control (Chih, Jan, Shu, & Lue, 2010). Factors related to school, friends and families affect the development of self‐efficacy in children. Generally, families with better economic, human or non‐material and social resources offer richer experiences to children, increasing their self‐efficacy (Schunk & Meece, 2005). Adolescents with high self‐efficacy are better able to bear the challenges of growing up (Schunk & Meece, 2005) and living with a long‐term condition (Resnick, 2014).

Self‐efficacy influences adolescents’ life, self‐management and diabetes outcomes. Self‐efficacy has been found to correlate significantly with positive outcome expectations, diabetes self‐management (Newton & Ashley, 2013) and quality of life (Cramm, Strating, Roebroeck, & Nieboer, 2013; Newton & Ashley, 2013). Adolescents, especially boys, with higher self‐efficacy have a higher probability of achieving target metabolic control (Chih et al., 2010).

Bandura’s (1977) concept of self‐efficacy is associated with empowerment, but it is not obvious as to whether self‐efficacy should be considered as an outcome or a precursor of empowerment (Aujoulat et al., 2007). The focus of this study is on self‐efficacy as part of empowerment of adolescents living with T1DM. Empowerment can be seen as a process where people gain abilities to control their lives (Gibson, 1991). Supporting empowerment represents a patient‐centred approach of care (Aujoulat et al., 2007; Croom et al., 2011) where healthcare professionals’ expertise in diabetes is combined with patients’ expertise in their own lives (Anderson & Funnell, 2010). In addition to the management of diabetes self‐care, adolescents need to be supported and helped to discover and develop the essential abilities they need to be responsible for their own lives with a long‐term condition (Funnell & Anderson, 2004). Empowerment can be promoted in nurse–patient relationship, and it comprises both the individual's internal process and a process between people (Aujoulat et al., 2007; Gibson, 1991).

Adolescents can become empowered when they have enough knowledge, skills and positive attitudes to make behavioural changes to improve their quality of life (Funnell et al., 1991). As a result of empowerment, patients can achieve a better sense of self‐efficacy concerning disease and treatment‐related behaviour (Anderson & Funnell, 2010; Aujoulat et al., 2007) as well as better metabolic control and quality of life (Anderson et al., 1995). The priorities and values in life may change. Patients are expected to self‐manage both the disease and their whole lives better (Aujoulat et al., 2007).

Diabetes needs to be integrated as part of one's own life (Karlsson, Arman, & Wikblad, 2008). However, individual differences exist in how and at what age children and adolescents assume responsibility for their diabetes management. Responsibility is gradually transferred from parents to adolescents. On the one hand, adolescents may succeed better in diabetes management and maintain good metabolic control when they receive positive support from parents and professionals (Anderson, 2009; Delamater et al., 2014; Karlsson et al., 2008). Adolescents can still be immature in decision‐making in diabetes management (Viklund & Wikblad, 2009). On the other hand, too early or full responsibility for diabetes care can increase anxiety, especially during puberty (Delamater et al., 2014). With sufficient support, the normal physical, emotional and cognitive development of the children and adolescents can be secured (Anderson, 2009). The Diabetes Association and diabetes outpatient clinics arrange education to adolescents to help everyday life with T1DM by increasing knowledge and abilities in self‐care of T1DM. Sharing emotions and experiences with a peer group is an important support on adolescents’ way to adulthood (Finnish Diabetes Association, 2018).

Adolescents’ psychosocial self‐efficacy is important given the large number of adolescents with T1DM in many countries, especially in Finland. There is a lack of studies assessing psychosocial self‐efficacy in adolescents, and as a promising valid and reliable instrument was available, the DES‐28 was selected. The aims of this study were to analyse the psychosocial self‐efficacy in adolescents with T1DM, to evaluate the associations between self‐efficacy and metabolic control and background variables and to determine the psychometric properties of the Finnish Diabetes Empowerment Scale (Fin‐DES‐28).

## 2 METHODS

### 2.1 Design, sample and settings

The study employed a descriptive, correlational survey design. The data were collected using a questionnaire consisting of background questions and the Fin‐DES‐28. The target group comprised all secondary school‐aged 13–16‐year‐old adolescents with T1DM (*N* = 189) in one (about 700,000 inhabitants) out of five university hospital districts in Finland. The number was confirmed by national statistics on reimbursements of medicine expenses for diabetes (KELA, 2013).

The sample size (*N* = 189) was estimated to be powerful enough to analyse the psychometric properties of the Fin‐DES‐28. The recommendations on the relation between items and respondents in validation studies vary from a ratio of 1:10–1:5. However, the minimum number of participants should be 100 respondents (Watson & Thompson, 2006). The target group of secondary school‐aged adolescents was selected because they bear a lot of responsibility for their diabetes care especially during the school day.

The study was carried out in one hospital district area, which included four outpatient clinics. According to national T1DM care guidelines, adolescents should have a meeting with a nurse specialist in diabetes care and a physician in the outpatient clinic every three months (Current Care Guideline for Diabetes in Finland, 2013). All eligible participants were to be reached at their regular clinic appointment. The inclusion criteria were as follows: the respondent is (a) 13–16 years old; (b) has been diagnosed with T1DM; (c) is Finnish speaking; (d) attends diabetes outpatient clinic appointments regularly; and (e) is able to independently fill in the questionnaire.

Exclusion criteria were as follows: the respondent is not Finnish speaking; is unable to fill in the questionnaire independently; has been diagnosed with type 2 diabetes; and not attending clinic appointments. The questionnaires were not planned to be sent home but to be completed at the outpatient clinics. The data were collected between February–May 2014.

Nurses in the outpatient clinics informed the eligible adolescents and their parents about the study at their regular appointments. A written information sheet was distributed both to adolescents and to their parents. Adolescents who agreed to participate in the study answered the questionnaire in the waiting room after the clinic visit. The questionnaires were sealed into envelopes and left in a collecting box in the outpatient clinic. In one out of the four clinics, it was requested that completed questionnaires sealed in envelopes be sent directly to the researchers. Glycated haemoglobin (HbA1c) was measured at every appointment (Current Care guideline for Diabetes in Finland, 2013). The diabetes nurse was asked to enter the latest HbA1c value in the questionnaire.

### 2.2 Instrument

The Diabetes Empowerment Scale (DES) was originally developed in the USA. It has been frequently used in adults with diabetes in many countries including Iceland (Sigurdardottir & Jonsdottir, 2008) and Iran (Mahjouri et al., 2012). In Hong Kong, Shiu et al. (2003) have developed the C‐DES‐20 and later a shortened version (C‐DES‐SF‐10) (Shiu et al., 2012). In Sweden, the DES has also been used with adolescents (Brorsson, Leksell, Viklund, & Lindholm‐Olinder, 2013; Hanberger et al., 2013; Viklund et al., 2007). Leksell et al. (2007) developed the Swe‐DES‐23, and Viklund et al. (2007) used it in an intervention study in adolescents with T1DM. Brorsson et al. (2013) used the instrument (Swe‐DES‐23) when examining parents and young people starting insulin pump therapy. Hanberger et al. (2013) applied the shortened version SWE‐DES‐SF‐10 to study the effect of the Diabit Web 2.0 net portal containing diabetes information and functions of the social network on empowerment of adolescents with diabetes.

The DES was developed to measure diabetes‐related psychosocial self‐efficacy in people with diabetes (Anderson et al., 2000). The pilot version of the DES was a 37‐item Likert‐type questionnaire. The structure of the DES was based on the earlier work of the study group in patient empowerment (Anderson, 1995; Anderson, Funnell, Barr, Dedrick, & Davis, 1991; Funnell et al., 1991). It had eight subscales, which were mainly derived from the four‐step behaviour change model based on counselling psychology: patient identification of problem areas, exploration of the emotions associated with those problems, development of a set of goals and strategies to overcome the barriers to achieving those goals and determining patients’ motivation to make a commitment to the behaviour change plan. Two subscales (Managing Stress and Obtaining Psychosocial Support) were added to the DES because these areas had been identified as major barriers and/or facilitators of behaviour change and psychosocial adaptation to diabetes (Anderson et al., 2000). The pilot version of the DES (Anderson et al., 1995) was used in a study (Anderson, Fitzgerald, Funnell, & Feste, 1997) and resulted in the current 28‐item (DES‐28) three‐subscale questionnaire, informed by factor analyses (Anderson et al., 2000).

The three DES‐28 subscales are as follows: Managing the Psychosocial Aspects of Diabetes (nine items), Assessing Dissatisfaction and Readiness to Change (nine items) and Setting and Achieving Diabetes Goals (10 items). The scale uses a 5‐point Likert format (1 = strongly disagree, 2 = disagree, 3 = neutral, 4 = agree and 5 = strongly agree). An overall score for the DES‐28 is obtained by adding all of the item scores and dividing the sum by 28. The higher the score, the higher the level of psychosocial self‐efficacy.

Internal consistency of the instrument has been found to be good. Cronbach's alpha coefficient of the original DES‐28 in a sample of adults was 0.96 (Anderson et al., 2000), and values of 0.84 for the Icelandic DES‐28 (Sigurdardottir & Jonsdottir, 2008), 0.89 for the Persian DES‐28 (Mahjouri et al., 2012), 0.91 for the Swedish DES‐23 (Leksell et al., 2007), 0.86 for the Chinese DES‐20 (Shiu et al., 2003) and 0.77 for the Chinese DES‐SF‐10 (Shiu et al., 2012) have been reported.

The test–retest reliability for the pilot version of the DES was 0.79. The same group of participants answered at the beginning and at the end of the 6‐week no‐treatment control period (Anderson et al., 2000). Test–retest reliability for the C‐DES‐20 was 0.75 evaluated with a 2‐week interval between tests in a subsample of 20 patients participating in the main study (Shiu et al., 2003).

The DES can be downloaded for research from the webpage of the Michigan Diabetes Research and Training Center (MDRTC). The right to use the DES was granted by MDRTC via email. The original instrument DES‐28 (Anderson et al., 2000) was translated into Finnish by standard forward–back translation (Sousa & Rojjanasrirat, 2011). The instrument was translated into Finnish by an authorized translator who has the right to carry out authorized translations after passing the translator examination. The translation was evaluated by the researchers and commented by two nurses with extensive experience in diabetes care. Based on the feedback, the tense used in the items and word choices were edited and the items were slightly shortened without changing the content. The instrument was then back‐translated into English by a second authorized translator. The correspondence between the two instruments in English was compared by the researchers. To ensure semantic and content equivalence, the translations were finally evaluated with a researcher who has used the DES instrument (Sigurdardottir & Jonsdottir, 2008).

In this study, socio‐demographic information including age, gender and family size was requested. In addition, we collected information about diabetes and its care, including the mode of diabetes treatment (injection therapy/insulin pump therapy), metabolic control using HbA1c (good <7.5%, moderate 7.5–8.5%, poor 8.6–10%, very poor >10%) (Koivisto, Knip, Nikkanen, & Saltevo, 1995), duration of diabetes (years, months), history of ketoacidosis (yes/no), inpatient updating education periods in the hospital (yes/no) and participation in courses offered by the Diabetes Association (yes/no). In addition to these, the DES has four background questions: (a) How often does your diabetes prevent you from doing normal daily activities? (b) How would you rate your understanding of diabetes and its treatment? (c) How able are you to fit diabetes into your life in a positive manner? and (d) How comfortable do you feel asking your doctor/nurse (added in this study) questions about diabetes? The response alternatives range from 1–7 (1 = never/poor and 7 = frequently/excellent).

### 2.3 Pilot study

The reliability and validity of the instrument, the clarity of the items and the feasibility of the scale were tested in a pilot study. The pilot study was carried out in a diabetes outpatient clinic for children and adolescents at a university hospital (*N* = 10) in January 2014. The adolescents filled in the questionnaires and were asked about the clarity of the items and for other comments to the questionnaire. Most of the feedback was positive, and the questionnaire did not require editing. The results of the pilot study were not statistically analysed and were not included in the main study.

### 2.4 Data analysis

The data were analysed using SPSS 21.0 software (IBM Corporation, Chicago, IL, USA). Descriptive statistics (frequencies, percentages, means and standard deviation) were used to describe the respondents and the study variables. Sum variables were formed from the items according to the theoretical structure of the instrument (Anderson et al., 2000).

Associations between the DES and its sum variables and HbA1c were calculated using the Spearman rank correlation coefficient. The Shapiro–Wilk test result showed that the DES was normally distributed. Therefore, the association of the DES with categorical independent variables (gender, mode of diabetes treatment, history of ketoacidosis, etc.) was tested with the *t* test (Grove, Burns, & Gray, 2013). To test associations between the DES and ordinal variables, the Spearman rank‐order correlation coefficient (rho) was used (LoBiondo‐Wood & Haber, 2014). Internal consistency of the items in the scale was assessed using Cronbach's alpha coefficients and item analysis (item to total, *r*) (DeVon et al., 2007). Significance level was set at *p* ≤ 0.05. In selecting statistical tests, an experienced statistician was consulted.

### 2.5 Ethical considerations

Research Ethics Committee approval was obtained from the Local Ethics Committee (22/2013). Chief administrators gave permissions to collect the data from outpatient clinics of the hospitals in the university hospital district area. The study was conducted according to the Declaration of Helsinki.

Both adolescents and parents were informed about the study both orally and using written information letters by nurses in the outpatient clinics. Participation in the study was voluntary, and adolescents had the right to refuse to participate in the study without it affecting their care or having to explain the reason for the refusal. Adolescents who agreed to participate in the study answered the questionnaire in the waiting room after the clinic visit. Answering the questionnaire was interpreted as informed consent. Responding was confidential and implemented without names, and no identifying information was asked. No extra invasive procedures were needed because HbA1c measurement is part of routine diabetes care. The questionnaires were stored in a locker, and the data were stored in the researcher's computer protected by a password.

## 3 RESULTS

### 3.1 Participants

A total of 65 adolescents participated in the study (response rate 34%). One questionnaire was rejected from the analysis because the respondent had left a message noting that he or she had answered the questionnaire without reading the items. There were slightly more girls (52%, *N* = 34) than boys among the respondents. According to national statistics on reimbursements of medicine expenses of diabetes, boys (56%) dominate over girls (44%) in the age group of adolescents aged 15–19 years in the hospital district (Kela, 2013).

The mean duration of diabetes was 7 years, ranging from 0.5–14 years. The mean of the glycated haemoglobin (HbA1c) was 8.5%, ranging from 6.1–11.9%. One fifth of the adolescents were in good metabolic control, most were in moderate (35%) or poor (40%) control, and 6% were in very poor control. One fifth of the adolescents had at some time been treated in hospital because of ketoacidosis, and nearly a third had been in hospital for inpatient updating education periods (Table 1).

**Table 1:** Demographic and diabetes‐related characteristics of the sample

Variable	N	%
Gender		
Female	34	52
Male	31	48
Diabetes treatment		
Injection therapy	35	58
Pump therapy	25	42
Metabolic control		
Good (<7.5%)	12	19
Moderate (7.5%–8.5%)	22	35
Poor (8.6%–10.0%)	25	40
Very poor (>10.0%)	4	6
Ketoacidosis		
Yes	13	20
No	52	80
Inpatient updating education periods in the hospital		
Yes	18	29
No	45	71
Diabetes Association courses		
Yes	39	60
No	26	40

### 3.2 The psychosocial self‐efficacy in adolescents

The mean value for Fin‐DES‐28_total_ was 3.88 (*SD* 0.45 on a scale from 1–5). The highest score of the subscales was in Managing the Psychosocial Aspects of Diabetes (mean: 3.99 *SD* 0.55), followed by Assessing Dissatisfaction and Readiness to Change (mean: 3.87 *SD*: 0.44) and Setting and Achieving Diabetes Goals (mean: 3.84 *SD*: 0.54) (Table 2).

**Table 2:** The Finnish Diabetes Empowerment Scale (Fin‐DES‐28)

Scale	*N*	*α*	Mean (*SD*) (range)
Fin‐DES‐28 total	60	0.93	3.88 (0.45) (3.04–4.82)
Managing the Psychosocial Aspects of Diabetes	65	0.89	3.99 (0.55) (2.44–5.00)
Assessing Dissatisfaction and Readiness to Change	61	0.75	3.87 (0.44) (3.00–4.89)
Setting and Achieving Diabetes Goals	63	0.89	3.84 (0.54) (2.50–5.00)

#### 3.2.1 Managing the psychosocial aspects of diabetes

The items with the highest scores were as follows: “know where I can get support for having and caring for my diabetes” (mean: 4.26 *SD*: 0.64) and “can ask for support for having and caring for my diabetes when I need” (mean: 4.26 *SD* 0.67). Almost all (90%) agreed or strongly agreed with the items.

The adolescents scored the following items lowest: “know the positive ways I cope with diabetes‐related stress” (mean: 3.69 *SD* 0.79) and “know what helps me stay motivated to care for my diabetes” (mean: 3.78 *SD* 0.78).

#### 3.2.2 Assessing dissatisfaction and readiness to change

In this subscale, the items with the highest scores were as follows: “can tell how I'm feeling about having diabetes” (mean: 4.14 *SD* 0.73) and “can tell how I'm feeling about caring for my diabetes” (mean: 4.12 *SD* 0.82). Most of adolescents (83%) agreed or strongly agreed.

Here, the lowest scored item was “know the negative ways I cope with diabetes‐related stress” (mean: 3.47 *SD* 0.73). Over half (53%) of the adolescents had no opinion (neutral), while only 42% agreed or strongly agreed. The second lowest scored item was “know what part(s) of taking care of my diabetes I am not ready to change” (mean: 3.49 *SD*: 0.80). Half (51%) of the adolescents agreed or strongly agreed.

#### 3.2.3 Setting and achieving diabetes goals

Adolescents scored the items “can reach my diabetes goals once I make up my mind” (mean: 4.22 *SD* 0.67) and “know which of my diabetes goals are most important to me” (mean: 4.14 *SD* 0.77) highest in this subscale. Most of the adolescents (89%, 82%) agreed or strongly agreed.

Adolescents scored lowest the item “can come up with good ideas to help me reach my goals” (mean: 3.55 *SD* 0.79). About half (49%) of the adolescents agreed or strongly agreed. The second lowest scored item was “can try out different ways of overcoming barriers to my diabetes goals” (mean: 3.62 *SD* 0.82). Over half (60%) of the adolescents agreed or strongly agreed.

#### 3.2.4 DES‐background questions

Most of the adolescents reported that diabetes did not prevent them from doing normal daily activities (never, 55%, *N* = 35). The adolescents experienced that they understood diabetes and its treatment well (response 5–7, 92%, *N* = 60). The adolescents said that they were able to fit diabetes into their lives in a positive manner (5–7, 86%, *N* = 56). The relationship between the adolescents and the doctor (5–7, 57%, *N* = 37) and the nurse (5–7, 56%, *N* = 36) was fairly good (Table 3).

**Table 3:** The background questions of the Diabetes Empowerment Scale and responses according to numbers 1–7 (1 = never/poor and 7 = frequently/excellent)

	*N*	1	2	3	4	5	6	7	*M*ean
		*N* (%)							
How often does your diabetes prevent you from doing normal daily activities	65	36 (55)	19 (29)	7 (11)	3 (5)	–	–	–	1.65
How would you rate your understanding of diabetes and its treatment	65	–	–	–	5 (8)	18 (28)	31 (48)	11 (17)	5.74
How able are you to fit diabetes into your life in a positive manner	65	–	1 (2)	2 (3)	6 (9)	15 (23)	31 (48)	10 (15)	5.58
How comfortable do you feel asking your doctor questions about diabetes	65	1 (2)	4 (6)	8 (12)	15 (23)	13 (20)	15 (23)	9 (14)	4.78
How comfortable do you feel asking your nurse questions about diabetes	65	1 (2)	3 (5)	8 (12)	17 (26)	8 (12)	16 (25)	12 (19)	4.91

### 3.3 The psychosocial self‐efficacy and background variables

No associations were found between psychosocial self‐efficacy and metabolic control or background variables. Psychosocial self‐efficacy as one main sum variable (mean value) was correlated with the background variables. The *t* test showed no statistically significant difference in psychosocial self‐efficacy (DES‐means) according to gender (*p* = 0.674), treatment mode (*p* = 0.493), ketoacidosis treatment (*p* = 0.725), inpatient education and revision periods in the hospital (*p* = 0.417) or attending Diabetes Association courses (*p* = 0.892).

The Spearman rank correlation coefficient showed no statistically significant associations between psychosocial self‐efficacy (DES‐means) and age (rho = −0.007, *p* = 0.958), family size (rho = −0.071, *p* = 0.593) or duration of diabetes (rho = 0.015, *p* = 0.913).

However, a positive association was found between psychosocial self‐efficacy and responses to DES‐background questions: understanding of diabetes and its treatment (rho = 0.420, *p* = 0.001), adjustment of diabetes to life (rho = 0.439, *p* < 0.001) and the relationship with the doctor (rho = 0.536, *p* < 0.001) and the nurse (rho = 0.522, *p* < 0.001) (Table 4).

**Table 4:** The background questions of the Diabetes Empowerment Scale and their correlation to the mean value of Fin‐DES‐28

Item	*N*	DES, rho
How often does your diabetes prevent you from doing normal daily activities	60	−0.123, *p* = 0.348
How would you rate your understanding of diabetes and its treatment	60	0.420, *p* = 0.001
How able are you to fit diabetes into your life in a positive manner	60	0.439, *p* < 0.001
How comfortable do you feel asking your doctor questions about diabetes	60	0.536, *p* < 0.001
How comfortable do you feel asking your nurse questions about diabetes	60	0.522, *p* < 0.001

Note

DES: Diabetes Empowerment Scale; rho: Spearman correlation.

### 3.4 Psychometrics

The Cronbach's alpha coefficient for Fin‐DES‐28_total_ was 0.93 and for the subscales from 0.75–0.89, indicating internal consistency. See Table 2 for Cronbach's alpha coefficient of Fin‐DES‐28 subscales, means, standard deviations and range. Item‐to‐total correlations ranged from 0.302–0.710 and correlations between sum variables from 0.258–0.781 (Tables 5, 6, 7). Correlations between two items of the subscale of Assessing Dissatisfaction and Readiness to Change were under general criterion (*r* < 0.3) (Sousa & Rojjanasrirat, 2011).

**Table 5:** Managing the psychosocial aspects of diabetes

Item/Sum variable	*N*	*M*	*SD*	Strongly agree (5), *N* (%)	Agree (4), *N* (%)	Neutral (3), *N* (%)	Disagree (2), *N* (%)	Strongly disagree (1), *N* (%)	Item to total, *r*
Managing the psychosocial aspects of diabetes (18, 20–27) In general, I believe that I:	65	3.99	0.55						
18. …know the positive ways I cope with diabetes‐related stress	65	3.69	0.79	10 (15)	27 (42)	27 (42)	–	1 (2)	0.694
20. …can cope well with diabetes‐related stress	65	3.94	0.83	16 (25)	33 (51)	12 (19)	4 (6)	–	0.648
21. …know where I can get support for having and caring for my diabetes	65	4.26	0.64	24 (37)	34 (52)	7 (11)	–	–	0.554
22. …can ask for support for having and caring for my diabetes when I need it	65	4.26	0.67	24 (37)	35 (54)	5 (8)	1 (2)	–	0.722
23. …can support myself in dealing with my diabetes	65	3.98	0.70	14 (22)	37 (57)	13 (20)	1 (2)	–	0.637
24. …know what helps me stay motivated to care for my diabetes	65	3.78	0.78	11 (17)	32 (49)	19 (29)	3 (5)	–	0.571
25. …can motivate myself to care for my diabetes	65	3.80	0.83	14 (22)	27 (42)	21 (32)	3 (5)	–	0.781
26. …know enough about diabetes to make self‐care choices that are right for me	65	4.17	0.82	23 (35)	34 (52)	5 (8)	2 (3)	1 (2)	0.587
27. …know enough about myself as a person to make diabetes care choices that are right for me	65	4.06	0.68	15 (23)	41 (63)	7 (11)	2 (3)	–	0.663

Note

*M*: mean; *N*: number; *r*: correlation coefficient; *SD*: standard deviation.

**Table 6:** Assessing dissatisfaction and readiness to change

Item/Sum variable	*N*	*M*	*SD*	Strongly agree (5), *N* (%)	Agree (4), *N* (%)	Neutral (3), *N* (%)	Disagree (2), *N* (%)	Strongly disagree (1), *N* (%)	Item to total, *r*
Assessing dissatisfaction and readiness to change (1–4,15–17,19,28) In general, I believe that I:	61	3.87	0.44						
1. …know what part(s) of taking care of my diabetes that I am satisfied with	65	4.09	0.68	18 (28)	35 (54)	12 (19)	–	–	0.536
2. …know what part(s) of taking care of my diabetes that I am dissatisfied with	64	4.00	0.74	14 (22)	39 (61)	8 (13)	3 (5)	–	0.459
3. …know what part(s) of taking care of my diabetes that I am ready to change	65	3.75	0.87	11 (17)	32 (49)	19 (29)	1 (2)	2 (3)	0.288
4. …know what part(s) of taking care of my diabetes that I am not ready to change	63	3.49	0.80	5 (8)	27 (43)	26 (41)	4 (6)	1 (2)	0.258
15. …can tell how I'm feeling about having diabetes	65	4.14	0.73	21 (32)	33 (51)	10 (15)	1 (2)	–	0.569
16. …can tell how I'm feeling about caring for my diabetes	65	4.12	0.82	22 (34)	32 (49)	9 (14)	1 (2)	1 (2)	0.466
17. …know the ways that having diabetes causes stress in my life	65	4.06	0.73	17 (26)	37 (57)	9 (14)	2 (3)	–	0.385
19. …know the negative ways I cope with diabetes‐related stress	64	3.47	0.73	6 (9)	21 (33)	34 (53)	3 (5)	–	0.414
28. …am able to figure out if it is worth my while to change how I take care of my diabetes	65	4.00	0.79	17 (26)	34 (52)	11 (17)	3 (5)	–	0.535

Note

*M*: mean; *N*: number; *r*: correlation coefficient; *SD*: standard deviation.

**Table 7:** Setting and achieving diabetes goals

Item/Sum variable	*N*	*M*	*SD*	Strongly agree (5), *N* (%)	Agree (4), *N* (%)	Neutral (3), *N* (%)	Disagree (2), *N* (%)	Strongly disagree (1), *N* (%)	Item to total, *r*
Setting and achieving diabetes goals (5–14) In general, I believe that I:	63	3.84	0.54						
5. …can choose realistic diabetes goals	64	3.95	0.70	14 (22)	33 (52)	17 (27)	–	–	0.611
6. …know which of my diabetes goals are most important to me	64	4.14	0.77	22 (34)	31 (48)	9 (14)	2 (3)	–	0.472
7. …know the things about myself that either help or prevent me from reaching my diabetes goals	65	3.89	0.79	15 (23)	30 (46)	18 (28)	2 (3)	–	0.667
8. …can come up with good ideas to help me reach my goals	65	3.55	0.79	8 (12)	24 (37)	29 (45)	4 (6)	–	0.603
9. …am able to turn my diabetes goals into a workable plan	65	3.69	0.87	11 (17)	28 (43)	22 (34)	3 (5)	1 (2)	0.681
10. …can reach my diabetes goals once I make up my mind	65	4.22	0.67	22 (34)	36 (55)	6 (9)	1 (2)	–	0.621
11. …know which barriers make reaching my diabetes goals more difficult	65	3.92	0.82	15 (23)	34 (52)	12 (19)	4 (6)	–	0.440
12. …can think of different ways to overcome barriers to my diabetes goals	65	3.63	0.78	8 (12)	29 (45)	24 (37)	4 (6)	–	0.712
13. …can try out different ways of overcoming barriers to my diabetes goals	65	3.62	0.82	8 (12)	30 (46)	21 (32)	6 (9)	–	0.664
14. …am able to decide which way of overcoming barriers to my diabetes goals works best for me	65	3.71	0.82	11 (17)	28 (43)	22 (34)	4 (6)	–	0.773

Note

*M*: mean; *N*: number; *r*: correlation coefficient; *SD*: standard deviation.

## 4 DISCUSSION

The mean for Fin‐DES‐28_total_ was 3.88, with subscales ranging from 3.84–3.99. These results suggest that the psychosocial self‐efficacy of the adolescents was at quite a good level while metabolic control was moderate or poor in most participants. We found no association between psychosocial self‐efficacy and metabolic control. This result differs from earlier studies (Chih et al., 2010; Newton & Ashley, 2013) where an association was found between adolescents’ self‐efficacy and their metabolic control. However, these studies used other instruments to measure self‐efficacy. There was a weak correlation between psychosocial self‐efficacy and metabolic control in one study in adults (Leksell et al., 2007) but not in another (Shiu et al., 2003). According to database searches, there have been no earlier studies on the association between self‐efficacy and metabolic control in adolescents measured with the DES. No associations were found between psychosocial self‐efficacy and metabolic control or background variables, but a positive association was found between psychosocial self‐efficacy and understanding of diabetes and its treatment, adjustment of diabetes to life and the relationship with the doctor and the nurse (DES‐background questions).

Although the average psychosocial self‐efficacy was at quite a good level, there were individual adolescents who needed more psychosocial support. In clinical practice, the DES could be used to target those in need of psychosocial support; this might assist clinicians to use the limited time for such support more effectively. However, a 28‐item scale may be too long to use during the health encounter; the short Swedish version (SWE‐DES‐SF‐10) looks promising (Hanberger et al., 2013) but requires testing in Finland. Specific items of the DES could also be used to guide the conversation with adolescents. A randomized controlled trial in diabetes care used the participants’ responses to knowledge, self‐care, empowerment and distress instruments to guide the intervention (Sigurdardottir et al., 2009). Further research with a larger sample is needed to test the feasibility and acceptability of the measure in adolescents. The DES was developed for adults with diabetes, which needs to be taken into account when considering how sensitive this instrument is in adolescents who may still be immature in decision‐making in diabetes management.

Mean HbA1c value was 8.5%. Only one fifth of the adolescents were in good metabolic control (HbA1c < 7.5%). The HbA1c level of the studied adolescents was a little higher than in an international study where one third (30%) of the adolescents (<15 years) were in good metabolic control (McKnight et al., 2015). The hormonal changes and developmental challenges of puberty can affect achieving good metabolic control (Tfayli & Arslanian, 2007). Virtual services offering information about self‐care and support for self‐monitoring can provide support for living with a long‐term illness. Client‐oriented digital healthcare services (The Virtual Hospital 2.0) have been recently developed in Finland, and the virtual house for children and adolescents with T1DM and their families will be being launched as well in the near future. The virtual house will consist of digital treatment pathways such as treatment of T1DM for newly diagnosed children and adolescents and the insulin pump therapy and other digital services supporting self‐care of T1DM (Virtual Hospital 2.0, 2018).

The results from this study support the internal reliability of the Fin‐DES‐28, with Cronbach's alpha coefficient of 0.93 for the total scale and 0.75–0.89 for the subscales. The psychometric properties of the Fin‐DES‐28 should be tested with a larger sample of Finnish adolescents.

### 4.1 Limitations and strengths

The STROBE reporting guidelines were used to ensure that all relevant information is included. Missing data were not imputated, and therefore, the number of responses vary. There were some limitations in this study, which need to be considered when interpreting the results. Due to the low response rate, the results are tentative and cannot be generalized extensively to the target group. In this study, girls dominated slightly over boys in the sample, which is in contradiction to the gender distribution of the adolescents reported to receive reimbursement for insulin in the area (KELA, 2013). Thus, there were more boys who did not participate in the study.

The participants were recruited from all four outpatient clinics of one out of five university hospital districts. The data collection period was long enough to catch all the potential adolescents during their regular 3‐monthly follow‐up visits (Current Care guideline for Diabetes in Finland 2013), but unwillingness to participate in the study was apparent. Thus, a longer period of data collection time would have been possible. However, as the three‐month period was used to include all adolescents with T1DM, extending the data collection period would have resulted in a situation where the same adolescents had their next encounters. The adolescents may have had low motivation to answer the questionnaire. Participants were not given any incentive for participation. Also, the timing of filling the questionnaire after the clinic visit may not have been ideal. Questionnaires were not planned to be given to be taken home as it was thought that they would not be returned. This fear proved to be relevant as in one of the four clinics the participants were allowed to take the questionnaires (*N* = 8) home to be mailed back and none of the questionnaires were returned.

## 5 CONCLUSIONS

Adolescents with T1DM self‐assessed their psychosocial self‐efficacy to be at quite a good level. However, the mean of glycated haemoglobin (HbA1c) in the sample was 8.5% (range 6.1%–11.9%), indicating moderate or poor metabolic control in most of the group. Interestingly, no associations were found between the psychosocial self‐efficacy measured with the Fin‐DES‐28 and the glycated haemoglobin level. The psychosocial self‐efficacy was not a good indicator of metabolic control in our study. The hormonal changes related to puberty and the developmental challenges of youth may affect the achievement of good metabolic control. The findings of a positive association between the psychosocial self‐efficacy and DES‐background questions, understanding of diabetes and its treatment, adjustment of diabetes to life and the relationship with the doctor and the nurse are important. The psychosocial self‐efficacy could be promoted by increasing adolescents’ understanding of diabetes and its treatment, adjustment of diabetes to life and the good relationship with the doctor and the nurse where adolescents can easily ask questions about diabetes.

In the future, there is a need to analyse other factors that help adolescents achieve better metabolic control. Virtual services might help some adolescents to self‐manage their diabetes better and to achieve better metabolic control as well. Adolescents are not very keen on filling questionnaires, which is why more innovative ways of evaluation would be welcome. Adolescents might also be more motivated by questionnaires in electronic form.

## RESEARCH ETHICS COMMITTEE APPROVAL

Research Ethics Committee approval was obtained from the Ethics Committee of the University of Turku (22/2013).

## AUTHOR CONTRIBUTIONS

AS and RS: Study conception/design. AS: Data collection/analysis. AS, RS and SS: Drafting of manuscript. AKS, SS and KN‐S: Critical revisions for important intellectual content. RS, SS, AKS and KN‐S: Supervision. AS in consultation with statistician Jouko Katajisto: Statistical expertise. RS and KN‐S: Administrative/technical/material support.
